# Growth Factors and Their Application in the Therapy of Hereditary Neurodegenerative Diseases

**DOI:** 10.3390/biomedicines12081906

**Published:** 2024-08-20

**Authors:** Shaza Issa, Haidar Fayoud, Alisa Shaimardanova, Albert Sufianov, Galina Sufianova, Valeriya Solovyeva, Albert Rizvanov

**Affiliations:** 1 Department of Genetics and Biotechnology, St. Petersburg State University, 199034 St. Petersburg, Russia; shaza.issa98@outlook.com (S.I.); haidar.fayoud@gmail.com (H.F.); 2 Institute of Fundamental Medicine and Biology, Kazan Federal University, 420008 Kazan, Russia; alisashajmardanova@kpfu.ru (A.S.); vavsoloveva@kpfu.ru (V.S.); 3 Department of Neurosurgery, Sechenov First Moscow State Medical University of the Ministry of Health of the Russian Federation (Sechenov University), 119991 Moscow, Russia; sufianov_a_a@staff.sechenov.ru; 4 The Research and Educational Institute of Neurosurgery, Peoples’ Friendship University of Russia (RUDN), 117198 Moscow, Russia; 5 Department of Pharmacology, Tyumen State Medical University, 625023 Tyumen, Russia; sufarm@mail.ru; 6 Division of Medical and Biological Sciences, Tatarstan Academy of Sciences, 420111 Kazan, Russia

**Keywords:** growth factors, hereditary neurodegenerative diseases, gene therapy, BDNF, GDNF, IGF-1, VEGF, NGF, FGF-2, CNTF, TGF-β1, HGF

## Abstract

Hereditary neurodegenerative diseases (hNDDs) such as Alzheimer’s, Parkinson’s, Huntington’s disease, and others are primarily characterized by their progressive nature, severely compromising both the cognitive and motor abilities of patients. The underlying genetic component in hNDDs contributes to disease risk, creating a complex genetic landscape. Considering the fact that growth factors play crucial roles in regulating cellular processes, such as proliferation, differentiation, and survival, they could have therapeutic potential for hNDDs, provided appropriate dosing and safe delivery approaches are ensured. This article presents a detailed overview of growth factors, and explores their therapeutic potential in treating hNDDs, emphasizing their roles in neuronal survival, growth, and synaptic plasticity. However, challenges such as proper dosing, delivery methods, and patient variability can hinder their clinical application.

## 1. Introduction

The term “Growth factors” (GFs), which is sometimes used as an alternative description of some cytokines, refers to a group of naturally occurring substances that present as either secreted proteins or steroid hormones [1]. Different characteristics can be used to categorize GFs into families, such as their structure and molecular organization, the nature and location of their receptors, and the signaling cascades they activate [2]. A general classification divides them into the following families: the platelet-derived growth factor (PDGF) family, the vascular endothelial growth factor (VEGF) family, the epidermal growth factor (EGF) family, the fibroblast growth factor (FGF) family, the insulin family, the hepatocyte growth factor (HGF) family, the neurotrophin family, the ephrin family, the agrin family, the glial cell line-derived neurotrophic factor (GDNF) family, and the angiopoietin family [3]. Another classification divides GFs into two main groups, according to their receptors’ locations: the membrane-receptor binding family and the intracellular-receptor binding family [2].

Having certain tissue-specificity, different GFs can be identified in distinct tissues [4], including epithelial [5], lymphoid [6], nervous [7], muscular [8], bone [9], and many other tissues. GFs use cellular receptors to regulate a wide variety of cellular processes on both intercellular and intracellular levels [2], including cell proliferation and survival [10], cell growth and differentiation [11], cell motility [12], metabolism regulation [13], and other functions that will be discussed later in this article. Having such features has made GFs an appealing option to be used in various therapeutic approaches targeting a range of diseases [14,15,16]. In particular, the potential of GFs in the treatment of hereditary neurodegenerative diseases (NDDs) is increasingly recognized in conditions like Alzheimer’s disease (AD), Parkinson’s disease (PD), and Huntington’s disease (HD), among others, which also will be discussed throughout the article.

## 2. Main Characteristics and Functions of Growth Factors

GFs show a scope of distinct characteristics that contribute to their different cellular functions. To begin, GFs’ ability to trigger a cascade of intracellular signaling events, following receptor binding, leads to the activation of key elements that alter gene expression patterns and induce cell cycle entry [17]. Therefore, having cell receptors, mostly with tyrosine kinase activity domain, facilitates the role of GFs in cell proliferation [18]. The activation of an intracellular cascade following GF receptor binding is also known to enable cell differentiation in different scenarios, including stem cell activation [19], the induction of lineage commitment [20,21], and morphogenesis control [22,23]. GFs also play a role in cell survival [24,25] by activating survival pathways and/or controlling apoptotic factors [26,27] or via GF-dependent DNA repair [28]. Furthermore, by inducing endothelial cell activation, proliferation, migration, and tube formation, GFs such as PDGF, VEGF, and FGF play important roles in the angiogenesis process [29,30]. Such characteristics of growth factors render them key elements in a variety of therapeutic approaches targeting, for example, myocardial infarction [31], wound healing [32], and anti-cancer therapy [33]. By regulating immune cell development, activation, and migration, GFs can also modulate immune responses, making GFs, again, valuable targets for therapeutic interventions aimed at enhancing immunity or controlling immune-related disorders [34,35]. FGF, VEGF, insulin-like growth factor (IGF), and multiple other GFs play important roles in metabolism regulation by affecting cell growth and differentiation, insulin resistance, lipid and energy metabolism, and glucose uptake [36,37,38]. The involvement of GFs in physiological processes, such as folliculogenesis and ovulation [39,40], implantation [41,42], and fetal growth [43], is yet further evidence of their significant regulatory functions.

## 3. Overview of Hereditary Neurodegenerative Diseases (NDDs)

NDDs encompass a spectrum of over 600 heterogenous conditions with a progressive nature, severely compromising both the cognitive and motor abilities of patients [44,45]. A defining feature of hereditary NDDs (hNDDs) is their underlying genetic component, with single or multiple genes and mutations contributing to disease risk, creating a complex genetic landscape [46]. For instance, among genetic mutations linked to known hNDDs, examples include *HTT* gene mutation in HD [47], *SOD1* in amyotrophic lateral sclerosis [48], *PSEN1*, *PSEN2*, and *APP* in AD [49], *ARSA* gene mutation in metachromatic leukodystrophy (MLD) [50], and others. These diseases are characterized by late onset, usually slow clinical progression, and protein aggregates that are specific to each disease, accumulating in neurons and/or glia [45,51]. As different NDDs predominantly affect different regions of the nervous system, they present with a wide range of symptoms. In general, their manifestations mainly include cognitive impairment [52], behavioral changes [53], motor dysfunction [54], and progressive disability [55]. Considering all previously mentioned characteristics of hNDDs, an accurate diagnosis should include a combination of diagnostic tools, including clinical evaluation, biomarker testing, imaging studies, and genetic testing [56,57]. According to the World Health Organization, NDDs are estimated to be the second leading cause of death in developed countries, surpassing cancer-related deaths and second only to cardiovascular disease-related deaths [58,59]. Although research in the field of therapeutics is dynamic, to this day, hNNDs still lack universally accepted curative treatments [60]. The main challenges in treating hNDDs include the complexity of addressing widespread neuronal cell death, along with the limited regenerative capacity of the central nervous system (CNS) and the limited capacity of most drugs to bypass the blood–brain barrier (BBB) [61]. Among current experimental therapeutic approaches are regenerative stem cell therapy [61,62], immunotherapy [63,64], gene therapy, including adeno-associated virus (AAV)-based, RNAi-based, alternative silencing strategies, and gene editing (CRISPR) [65,66,67], in addition to non-pharmacological interventions, such as cognitive rehabilitation and speech therapy [68]. Although these therapeutic approaches aim to improve the quality of life for patients and, in some cases, prevent further deterioration, they do not offer complete recovery or the restoration of degenerated neural structures [59].

## 4. Growth Factors in the Therapy of Hereditary Neurodegenerative Diseases

**Figure 1:**
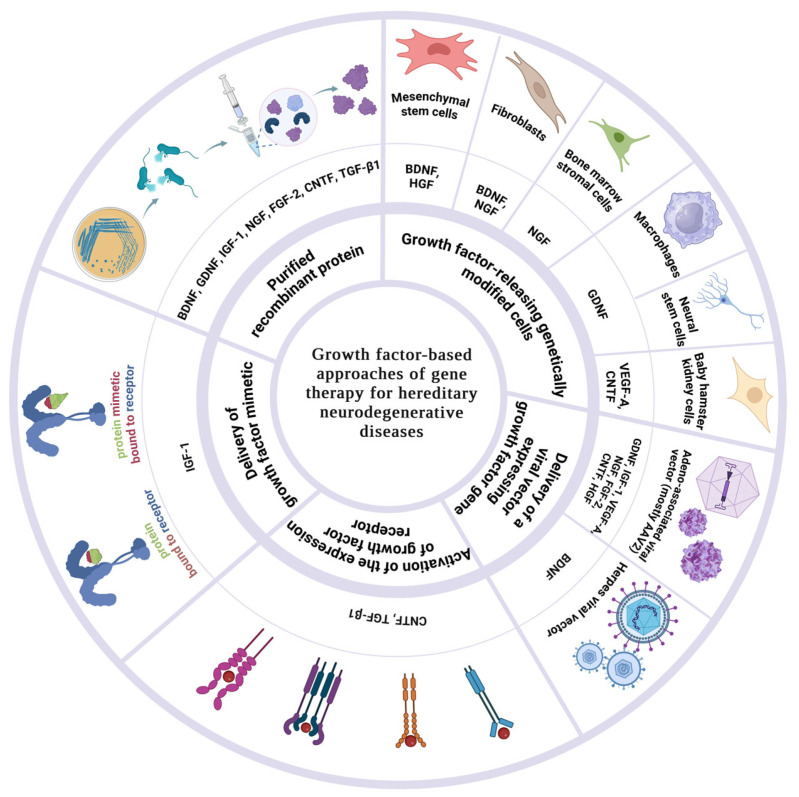
This figure summarizes GF-based gene therapy approaches for hNDDs covered in this review. Purified Recombinant Protein: This approach involves obtaining and directly administering purified recombinant GFs (e.g., BDNF, GDNF, IGF-1, NGF, FGF-2, CNTF, and TGF-β1) to the target cells. Growth Factor-Releasing Genetically Modified Cells: involves the utilization of cells (e.g., mesenchymal stem cells, fibroblasts, bone marrow stromal cells, macrophages) genetically engineered to release GFs like BDNF, HGF, NGF, and GDNF. Delivery of Growth Factor Gene: This involves the delivery of genes encoding GFs directly to target areas using viral vectors (e.g., AAV and HSV) in order to enable the patient’s own cells to produce the necessary GFs, such as BDNF, GDNF, VEGF-A, and CNTF, providing a sustained therapeutic effect. Activation of the Expression of Growth Factor: Using pharmacological agents or small molecules to enhance the endogenous expression of GFs (e.g., CNTF and TGF-β1) within the target cells. Delivery of Growth Factor Mimetic: Employing mimetics, i.e., synthetic molecules designed to mimic the biological activity of natural GFs. These mimetics bind to and activate GF receptors, such as IGF-1 mimetics, providing neuroprotective effects similar to endogenous factors.

Having distinct mechanisms of action, GFs engage in the restoration, protection, and generation of neurons and their functionality [69]. Moreover, they enable unique receptor activation that, despite their short half-life, initiates cascades of reactions, resulting in the activation of transcription factors, with effects that can last for extended periods [70]. GFs also contribute to enhanced synaptic plasticity, aiding in the maintenance of neural connectivity, which may be disrupted in hNDDs [71]. Some endogenous repair mechanisms can be activated by GFs following an injury to the CNS [72]. Considering all these characteristics of GFs, coupled with their anti-inflammatory effects and trophic support to cells, GFs play a significant role in the contest of therapy for hNDDs, provided appropriate dosing and safe delivery approaches are ensured [69,73]. The following section of this article delves into a more detailed exploration of GFs with neurotrophic effects that can be applied in the therapy of hNDDs. Table 1, Table 2, Table 3, Table 4, Table 5, Table 6, Table 7, Table 8 and Table 9 summarize GFs’ applications in therapy for different hNDDs, and Table 10 summarizes their different characteristics. Figure 1 summarizes GF-based gene therapy approaches for hNDDs covered in this review.

### 4.1. Brain-Derived Neurotrophic Factor (BDNF)

BDNF is a GF belonging to the neurotrophin family that was discovered and purified from pig brains by Barde et al. in 1982 [189]. Despite the considerable sequence homology and processing shared by many other neurotrophins, BDNF has become the most studied neurotrophin since its discovery due to its diverse roles in the CNS [190]. This highly conserved protein is primarily synthesized as an inactive precursor (28–32 kDa), which undergoes proteolytic cleavage to form biologically active mature BDNF (mBDNF) comprising 247 amino acids [191,192]. The primary source of BDNF secretion is neurons and glial cells, and it is prevalent in the cortical region, hippocampus, and visual cortex, beside other areas of the CNS [135,193]. BDNF binds its high-affinity tyrosine kinase receptor B (TrkB), as well as the low-affinity p75 neurotrophin receptor (p75^NTR^), initiating various intracellular signaling cascades that play crucial roles in neuronal survival, growth, and synaptic plasticity [192]. By binding its TrkB receptor, BDNF can induce gene expression by activating transcription factors, such as CREB, contributing to the modulation of neuronal function, plasticity, and survival [194,195]. Accordingly, BDNF has been linked to a wide range of physiological functions in the CNS, including neuronal survival 8 [134], neuroplasticity and synaptic plasticity [135], neurogenesis and synaptogenesis [136], cognitive function [137], dendritic branching [138], regulation of gene expression [135], and modulation of excitatory and inhibitory neurotransmitter profiles [134]. This broad spectrum of functions highlights the importance of BDNF in the nervous systems and thereby implicates its dysregulation in various hNDDs, establishing this GF as a crucial tool in therapeutic approaches for many of these conditions.

For instance, BDNF has been explored as a potential therapeutic target for HD, where a mutation in the *HTT* gene leads to neuronal atrophy, resulting in progressive motor dysfunction, cognitive decline, and ultimately, severe disability [196]. Notably, a significant reduction in BDNF levels has been observed in the affected brain regions of HD patients, accompanied by substantial alterations in TrkB receptors [197]. Accordingly, in vivo research by Pollock et al. in 2016 investigated the therapeutic potential of BDNF delivered through mesenchymal stem/stromal cells (MSC/BDNF) in HD mice [74]. Using two HD mouse models, the study found that MSC/BDNF treatment reduced striatal atrophy, alleviated anxiety, increased neurogenesis-like activity, and extended lifespan. Subsequent research was later proposed by the authors to build on the promising outcomes of using MSC/BDNF in HD [74]. They expressed their intention to submit an investigational new drug application to the FDA for a future phase I safety and tolerability trial of MSC/BDNF in HD patients [198].

Spinal muscular atrophy (SMA) and amyotrophic lateral sclerosis (ALS) are other common forms of motor neuropathies, and BDNF has been suggested as being a possible therapeutic option [199]. ALS is caused by genetic mutations in *C9orf72*, *SOD1*, and *FUS*, while SMA is primarily caused by mutations in the *SMN1* gene [200,201]. However, despite their different genetic backgrounds, both diseases involve altered TrkB signaling and BDNF levels, along with motor neuron degeneration, leading to muscle weakness and atrophy [199]. A phase I/II trial conducted by Ochs et al. investigated the intrathecal delivery of recombinant BDNF for therapy in ALS patients [75]. The results showed that doses of up to 150 microg/day were well tolerated, with reversible CNS effects at higher doses. However, although patients reported mild sensory symptoms, the study’s design and the small patient sample did not allow for conclusions to be drawn about treatment efficacy [75].

In PD, another NDD primarily caused by the loss of dopamine-producing neurons in the brain [202], there is a notable decrease in BDNF serum levels [203]. Accordingly, BDNF is again regarded as an intriguing therapeutic target for PD, whether in the context of gene therapy approaches or non-invasive exercise training trials [204]. Since the 1990s, in vivo studies exploring BDNF therapeutic potentials in PD have steadily increased [205]. The key distinction between these studies lies in the timing of BDNF administration. When administered before PD induction, BDNF has shown promise in preventing neuronal loss and increasing the survival of dopaminergic neurons in the substantia nigra (SN) and their projections to the striatum (ST) [76,77]. However, in the case of post-PD induction, some studies found no recovery of dopaminergic neurons in SN [78].

Similarly, altered expression levels of BDNF have been observed in murine animal models of spinocerebellar ataxia type 1 (SCA1), which is a hNDD resulting from a mutation in the *ATXN1* gene, causing progressive motor deficits [206]. More specifically, in the later stages of SCA1, BDNF levels were observed to decline following an initial increase during the early stages, which may play a role in the occurrence of pathological degeneration [79]. In their in vivo research, Dr. Marija Cvetanovic’s team assessed the therapeutic potential of BDNF in an SCA1 murine model, both in the pre-symptomatic and post-symptomatic stages [79,207]. The findings indicated that BDNF could delay the onset of motor impairments and neuronal degeneration, as well as ameliorate them in the post-symptomatic stage. However, BDNF alone was not found to affect gene expression changes in Purkinje cells; instead, it should be combined with additional gene therapy strategies.

Another prevalent type of ataxia, where the therapeutic potential of BDNF has been investigated, is Friedreich’s ataxia (FRDA), which is a hNDD caused by mutations in the *FXN* gene, causing motor dysfunction, as well as cardiopathies [80]. Both in vitro and in vivo studies have demonstrated the neuroprotective potential of BDNF in the context of FRDA. In vitro studies focused on FXN-deficient neurons showed that BDNF prevented apoptosis triggered by *FXN* gene knockdown [80]. Additionally, in vivo experiments using a murine model of FRDA revealed that BDNF not only prevented apoptosis but also hindered the development of neuropathology characteristic of FRDA [81]. In silico research has also highlighted BDNF as a potential therapeutic target in FRDA [208]. More specifically, miRNA-10a-5p was identified as a negative regulator of BDNF, and accordingly, correcting FRDA cells resulted in a decrease in miRNA-10a-5p and an increase in BDNF levels. This suggests a potential enhancement in neuronal growth associated with the upregulation of BDNF [208].

### 4.2. Glial Cell Line-Derived Neurotrophic Factor (GDNF)

GDNF is another neurotrophic factor, and it belongs to the transforming growth factor beta (TGF-β) superfamily [209]. GDNF was first discovered in 1993 in rat B49 glial cell lines and was characterized as a novel neurotrophic factor with specific activity on dopaminergic neurons [210]. Accordingly, GDNF has since been the focus of numerous studies investigating its mechanisms of action and therapeutic potential, especially for PD, where dopaminergic neurons are predominantly affected [211,212,213]. GDNF primarily binds to a receptor complex consisting of Ret receptor tyrosine kinase and one of a number of glycosylphosphatidylinositol (GPI)-anchored cell surface proteins, such as GFRα1–4 [214]. When binding to its RET receptor, GDNF initiates a complex series of intracellular signaling events that contribute to a wide variety of functions both inside the nervous system and outside. For instance, several studies have established GDNF as a potent survival factor for different neuronal populations, including motor and sensory neurons [139], midbrain dopamine neurons and noradrenergic neurons [140], and others. Moreover, GDNF has been found to control the migration and neuronal differentiation in the enteric nervous system in vivo [141]. Other main functions of GDNF outside the nervous system include the regulation of ureteric budding and branching during kidney development [142] and spermatogenesis regulation [143]. Similar to BDNF, the wide involvement of GDNF in nervous system functions has made it a key player in both normal neurobiology and as a therapeutic target for hNDDs.

Primarily, the potential of GDNF has been explored in the context of PD. As mentioned above, the demonstrated neuroprotective effects of GDNF on dopaminergic neurons have made it a promising candidate for slowing or preventing degeneration in PD. Accordingly, since its discovery, a series of in vivo studies and clinical trials have investigated infusions or gene delivery of GDNF in PD patients [84,85,86,87]. Although none of the trials have provided robust and consistent evidence for clinical efficiency, GDNF has generally shown a favorable safety profile, which is a crucial factor when considering its use as a long-term treatment option [88,89,90,91]. Challenges in these trials include dosing, delivery route, and immune response for the recombinant protein [215]. The inconsistency in the outcomes of trials prompts further investigation to understand the factors influencing the results, especially considering the potential neuroprotective and regenerative effects demonstrated in vivo. For instance, in a rodent animal model, the systemic delivery of GDNF–macrophages has shown promising results for both early and late stages of the disease. This includes enhanced brain tissue integrity, the restoration of most motor functions, sustained therapeutic effects, reduced neuroinflammation, and diminished α-synuclein aggregation [82]. In non-human primates, behavioral improvement and enhancements in motor function have also been observed [216], as well as the improved protection of dopaminergic neurons and higher dopamine levels following AAV-mediated GDNF gene delivery [83].

After the potential of GDNF was initially explored in relation to PD, the research was extended to HD. Similarly, preclinical studies in rodent animal models demonstrated that GDNF cell and gene therapy could protect and promote the survival of neurons and significantly enhance performance in motor tasks and neurological assessments, which was also supported by histological analyses showing higher neuronal counts in the targeted brain regions [92,93].

### 4.3. Insulin-Like Growth Factor 1 (IGF-1)

IGF-1, a peptide weighing 7.6 kDa and composed of 70 amino acids, stands out as a key member of the insulin-like growth factor family [217]. Discovered in 1957 and later isolated from human serum in 1976 by Rinderknecht and Humbel, IGF-1 shows a structural resemblance to insulin; however, it remains unaffected by insulin antibodies [218]. While hepatocytes primarily produce IGF-1, it is also synthesized in several brain regions, including the brain stem, cerebellum, cerebral cortex, and the striatum, highlighting its diverse physiological roles [219]. This GF is transported by IGF-binding proteins (IGFBPs) in the bloodstream to act on various target tissues [220]. IGF-1 binds with high affinity to the tyrosine-kinase IGF-1 receptor (IGF-1R) [221], facilitating diverse biological functions. These include growth stimulation [144], cellular proliferation and apoptosis [145], immunomodulatory functions [146], and CNS-related functions, such as neurogenesis, angiogenesis, neuroprotection, myelination, modulation of neuroinflammatory response, and neuroplasticity [222,223]. These properties make IGF-1 a GF of interest in research related to hNDDs both in vitro and clinically. 

Observations of disrupted IGF-1 signaling in AD patients, characterized by a reduced active/inactive IGF-1 ratio and elevated IGF-1R expression, prompted Selles et al. to explore the neuroprotective potential of IGF-1. Their in vivo study utilized a recombinant adenoviral vector (RAd-*IGF1*) to enhance IGF-1 expression in a murine model of AD. As a result, IGF-1 transduction was found to block memory impairment in AD mice, suggesting that enhancing IGF-1 expression in the brain could serve as a potential strategy against neuronal damage and memory loss in AD [94].

Nevertheless, trials exploring the potential application of IGF-1 for ALS and SMA revealed a lack of efficacy in patients. More specifically, a phase III, randomized, double-blind, placebo-controlled study investigated the subcutaneous delivery of IGF-1 for 2 years in ALS patients [95]. No significant improvement in outcomes was observed compared to the initial point, and IGF-1 therapy was found to be non-beneficial for this condition.

Similarly, a randomized, double-blinded, multicentered, and placebo-controlled study focused on spinal and bulbar muscular atrophy (SBMA) patients evaluated the safety, tolerability, and therapeutic potential of an IGF-1 mimetic with a longer half-life called BVS857 [96]. After 12 weeks, it was found to provoke an immune response, with no significant improvement being observed in strength or function of muscles, suggesting the need for further evaluation of this therapeutic option [96].

Furthermore, an ex vivo study was conducted by Luana et al. to investigate the effects of activating the IGF-1/insulin signaling pathway in lymphoblasts derived from HD patients [97]. The results revealed improved mitochondrial and metabolic function, as well as energy production, highlighting the role of IGF-1 in HD lymphoblasts [97]. This study was prompted by observations in previous research that suggested a correlation between high IGF-1 levels and cognitive decline, mainly in attention and executive function, in HD patients [224]. In the latter, the authors suggested that the correlation could be attributed to IGF-1 resistance, resulting in high IGF-1 levels with low IGF-1 effects.

In a clinical pilot study conducted by Sanz-Gallego et al., the safety, tolerability, and therapeutic potential of IGF-1 therapy were tested in FRDA patients over 12 months [98]. While a decrease in disease progression was observed during the therapy, some patients exhibited high scores on the ataxia scale towards the end of the study [98]. Accordingly, IGF-1 was found to significantly decrease FRDA progression but did not entirely prevent it [98].

Another form of ataxia in which IGF-1 has garnered attention, is ataxia telangiectasia (AT). AT is a hNDD caused by mutations in the *ATM* (ataxia telangiectasia mutated) gene, causing progressive cerebellar ataxia, telangiectasias, immunodeficiencies, heightened susceptibility to infections, and cancer predisposition [225]. Notably, a correlation has been identified between the deficiency of the IGF-1 axis and elevated ataxia scores, coupled with severe neurodegeneration in this disorder [99]. 

### 4.4. Vascular Endothelial Growth Factor A (VEGF-A)

VEGF-A is a member of the VEGF family, sharing a highly conserved cystine-knot motif structure with other family members [226]. The discovery of the first VEGF dates back to the 1980s, when Dvorak and colleagues initially identified it as a secretion from tumor cells, naming it vascular permeability factor (VPF) [227]. Subsequently, Ferrara and his team conducted further studies, leading to its reclassification as VEGF [227]. Different members of this family, including VEGF-A, VEGF-B, VEGF-C, VEGF-D, and Placental Growth Factor (PlGF), have distinct features, with VEGF-A being the most studied one, and thereby, often referred to as VEGF [228]. VEGF-A binds to VEGF Receptors (VEGFRs), mainly VEGFR1 and VEGFR2, initiating intracellular signaling pathways that play a pivotal role in various cellular processes [229]. VEGF-A is mainly known for its role in vasculogenesis, angiogenesis, and neuroprotection, among other functions [230,231]. Its main sources in the CNS include endothelial cells engaged in angiogenesis and maintaining barrier permeability, glial cells, and neurons [232]. Looking into VEGF-A functions in the nervous system in more detail, this GF has been found to enhance synaptic plasticity in vivo, thereby influencing memory and learning processes [147,148]. Through its involvement in angiogenesis and reducing neuroinflammation, VEGF-A has also been proven to support neuronal survival in vivo [149]. Accordingly, the upregulation of VEGF-A may counteract neuronal damage and enhance survival pathways [150]. The regenerative impact of VEGF-A has been proved in both the CNS and PNS (peripheral nervous system) [233]. More specifically, VEGF-A has been associated with enhanced axonal growth [151] and the activation of Schwann cells [152], crucial for the recovery and restoration of neural connectivity.

In the context of hNDDs, there has been lately growing interest in exploring the potential of VEGF-A. For instance, a prospective longitudinal study was conducted as part of the Alzheimer’s Disease Neuroimaging Initiative in order to assess the association between VEGF levels and brain aging outcomes over time in a group of individuals with varying cognitive statuses: normal, mild, and AD [100]. The outcomes suggested a link between high levels of VEGF in the cerebrospinal fluid (CSF) and a healthier pattern of brain aging, characterized by larger hippocampal measures and a slower decline in cognition and memory over time [100]. Additionally, this study investigated the interactions of VEGF with established AD biomarkers, highlighting that VEGF’s neuroprotective effects were particularly pronounced in the presence of enhanced AD biomarkers [100]. This implies that VEGF could serve as a more beneficial protective factor against neurodegeneration in individuals who exhibit early signs of AD. On the genetic level, several studies have found that the interaction between VEGF-A and Apolipoprotein E (APOE) ε4 allele, which is a genetic risk factor for AD, positively affects cognitive performance and could strongly influence the risk of developing AD [234,235,236]. 

Similarly, an observed correlation between low VEGF levels in plasma and ALS has highlighted the potential neurotrophic role of VEGF in motoneurons, suggesting promising therapeutic possibilities [237]. Based on this correlation, a recent in vivo study investigated the impact of VEGF on ALS progression via AAV-mediated delivery in a murine disease model [101]. The outcomes revealed significant delay of ALS in mice, preserved motor and neurological functions, and a longer life span, with the involvement of underlying factors, such as oxidative stress and autophagy, supporting the effects of VEGF [101].

AAV-mediated gene therapy was employed to explore the potential of VEGF in animal models of PD. These in vivo studies consistently reported neuroprotective effects on dopaminergic neurons and positive behavioral outcomes following VEGF delivery in PD murine models [102,103]. A study by zou et al., employing both in vitro and in vivo approaches, revealed that VEGF, influenced by CYS C, could be a potential therapeutic target for PD treatment [104]. In this experiment, the delivery of Cystatin C (CYS C) into the substantia nigra in a PD experimental murine model resulted in elevated VEGF in the targeted area, consequently contributing to the preservation of dopaminergic neurons [104]. Additionally, VEGF demonstrated a dual protective role by promoting angiogenesis in vitro, supporting both neuronal survival and vascular function [104]. Therefore, the research suggested the modulation of CYS C pathways involving VEGF could serve as a therapeutic strategy for PD. Another in vitro/in vivo study implemented a non-viral gene therapy approach for PD using a polymeric gene carrier to deliver the VEGF gene to dopaminergic neurons in a murine PD model [105]. As a result, VEGF-treated rats showed preserved motor function, with no loss of dopaminergic neurons in the targeted area [105]. The suppression of both microglial activation and apoptosis were another two properties proposed to contribute to the overall therapeutic effect of VEGF in this study.

### 4.5. Nerve Growth Factor (NGF)

NGF was the first identified member of the neurotrophin family [238]. It was discovered and purified from mouse sarcoma 180 by Levi-Montalcini and Cohen in 1957 [239], earning them the Nobel Prize in Physiology or Medicine in 1986 [240]. NGF is primarily synthesized as a 130 kDa precursor named ProNGF, which undergoes proteolytic cleavage to generate mature NGF [241]. These two forms have different biological activities and use different receptors. More specifically, ProNGF binds and activates p75 neurotrophin receptor (p75^NTR^), triggering inflammatory processes and apoptosis, a feature that has marked ProNGF as a potential therapeutic target for injuries to the nervous system [242]. Mature NGF mainly uses tyrosine kinase A receptor (TrKA) to initiate different signaling cascades to promote a wide spectrum of functions [241], and unlike its precursor, it has a pro-survival, anti-apoptotic role [243]. NGF has major roles both during development and in adulthood, including the modulation of neuronal growth, proliferation, activation, and survival [153], the regulation of sensory neuron differentiation [154], the modulation of perception [155], axonal target innervation [156], the maintenance of cholinergic neurons [157], the regulation of synaptic plasticity through areas of the limbic system and, subsequently, learning and memory abilities [158]. Moreover, lower levels of NGF have been associated with different psychiatric disorders, including depression [153].

Given its diverse range of functions, NGF has been studied in relation to many hNDDs. Notably, its role in stimulating cholinergic functions has led to extensive research in the context of AD [244]. Changes in NGF signaling have been identified to be one of the earliest events in AD pathology [158]. Supported by promising in vivo results, multiple clinical trials have investigated the therapeutic effects of NGF in the context of AD [245]. For instance, a controlled, double-blind, randomized, multicenter study evaluated intracerebral delivery of AAV2-NGF in AD patients [107]. Phase I of the study proved the feasibility and tolerance of AAV2-NGF, as well as the long-term production of biologically active NGF (up to 7 years) [106]. However, there were no changes in cognition abilities or clinical outcomes in patients after phase II [107], later attributed to the chosen delivery route, suggesting the use of an enhanced-delivery approach to overcome targeting issues [246]. A similar clinical trial evaluated the AAV-mediated delivery of NGF in AD patients and revealed a conserved ability of degenerating brain regions to interact with the delivered GF, as evidenced by axonal sprouting and the activation of cell signaling, with no NGF-related adverse effects being reported [108]. A phase I clinical trial conducted by Tuszynski et al. tested ex vivo NGF gene delivery in AD patients using genetically modified fibroblasts expressing NGF [247]. The outcomes demonstrated feasibility and slower disease progression with strong growth response following therapy. The encapsulated cell bio-delivery of NGF (NGF-ECB) has also been gaining interest for use in AD therapy [248,249]. A phase I clinical trial applied this approach to six AD patients, resulting in good tolerance in all patients, with three patients showing less brain atrophy and higher CSF cholinergic markers [109,110].

While NGF has garnered particular interest for its role in supporting cholinergic neurons in AD, it has also been investigated in other hNDDs. In a murine model of HD, the intracerebral delivery of NGF has also shown a considerable positive effect on cognitive function [111]. Following therapy, elevated cholinergic markers were noted in specific brain regions, along with restored neurogenesis in the hippocampus and enhanced spatial working memory in HD mice. Notably, lower plasma NGF levels in humans have also been found to correlate with disease intensity [250].

Similarly, in a murine model of PD, genetically modified bone marrow stromal cells (BMSCs) expressing NGF were tested, leading to induced neurogenesis and a significant improvement in the rotational behavior of treated animals [112]. Moreover, reduced CSF levels of NGF have been lately proposed as potential prognostic and diagnostic biomarkers for PD [251,252]. 

In ALS, the exact role of NGF is still not entirely understood; however, there have been multiple in vivo studies showing increased NGF levels in the spinal cord and astrocytes, with the latter promoting motor neuron degeneration [253,254]. Accordingly, NGF was hypothesized to play this pro-degenerative role in ALS through its p75^NTR^ receptor [255]. There has been one recent retrospective observational study testing the effect of mouse nerve growth factor (mNGF) combined with riluzole (a FDA-approved drug for ALS) on ALS patients. While the mNGF treatment was safe and well tolerated, it did not result in significant clinical changes in ALS progression, suggesting the need for further larger studies to establish more convincing outcomes [113].

### 4.6. Fibroblast Growth Factor-2 (FGF-2)

FGF-2, also known as basic FGF, or bFGF, is a member of the FGF family, which comprises a total of 23 factors [256]. The discovery of FGFs involved contributions from various researchers during the 1970s and 1980s [257]. In 1974, FGF-2 was purified from bovine pituitary glands by Gospodarowicz et al., and the human recombinant variant was later synthesized in 1988 [258]. The term ‘basic’ differentiates it from acidic FGF or FGF-1, which was discovered at the same time as FGF-2. This GF binds to an isoform of the FGF receptor (FGFR1–4), depending on cell type or tissue, in a heparan sulfate proteoglycan (HSPGs)-dependent manner [259]. FGF-2 exists in different isoforms, resulting from alternative translation, including low-molecular-weight (18 kDa) and high-molecular-weight (21–34 kDa) isoforms [260]. FGF-2, unlike most other FGFs, lacks a signal peptide, resulting in a non-classical secretion pathway [261]. Its main source in the CNS is astrocytes, from which it becomes dispersed across the cortex, hippocampus, and hypothalamus [167]. It is primarily recognized for its role in cell proliferation as it acts as a potent mitogen for different cell types [159], a feature that makes it frequently utilized as a common supplement in culture mediums [262]. In addition, FGF-2 plays an important role in the process of angiogenesis [160], facilitating tissue repair and regeneration, which is another pivotal function of FGF-2 [161]. By enhancing the activation and migration of different cell types, FGF-2 contributes to various processes, such as inflammation, wound healing, and even cancer progression and metastasis [162,163].

Beyond that, FGF-2 provides a wide spectrum of neurotrophic effects, among which is promoting neuronal survival, as evidenced by the anti-apoptotic role it plays on sensory neurons upon local application to the transected sciatic nerve in vivo [164]. Its previously mentioned role in cell proliferation extends to the nervous system via the induction of neurogenesis and neuronal maturation [165]. Moreover, FGF-2 promotes axonal growth and dendritic arborization, thereby contributing to the establishment of neuronal connectivity [166]. FGF-2 has also been shown to support synaptic plasticity, which translates into effects on cognition and memory [167].

Given its neurotrophic effects, the potential of FGF-2, and other members of its family, have been explored in the context of numerous hNDDs. For instance, in AD, the overexpression of FGF-2 has been reported to subsequently restore spatial learning, enhance synaptic connections, and neurogenesis [164]. More specifically, an in vivo study conducted by Katsouri et al. explored the effects of the subcutaneous delivery of recombinant FGF-2 into APP 23 transgenic mice. This study reported improved spatial memory, elevated levels of astrocytes in the hippocampus, and the potential modulation of inflammatory responses by reducing the expression of inducible nitric oxide synthase [114]. These results align with a previous study conducted by Kiyota et al., where AAV-mediated FGF-2 delivery into the hippocampi of a murine AD model resulted in improved spatial learning and long-term potentiation [115]. A summary of in vitro and in vivo studies exploring FGF-2 therapeutic potentials and effects for AD is provided in detail in a recent review article produced by Alam et al. [116].

Similarly, the neurotrophic effects of FGF-2 have also been relevant in PD research. A recent in vitro/in vivo study reported that silencing miR-497-5p, a microRNA upregulated in PD, improved motor symptoms, reduced apoptosis, and stimulated autophagy in a mouse model. These effects were mediated through the regulation of FGF2. These findings suggest that targeting miR-497-5p and its regulation of FGF2 could be a potential therapeutic strategy for PD management [117]. Another recent piece of research explored FGF2’s influence on the release of extracellular vesicles (EVs) in hippocampal neurons and its relevance to PD [118]. As a result, FGF-2 was found to enhance the release of EVs enriched with two key regulators implicated in membrane trafficking: Rab8b and Rab31 [118]. Accordingly, it was suggested that FGF2-induced Rab enrichment in EVs could play a role in molecular mechanisms related to non-motor symptoms in PD, such as hearing loss and dementia [118].

Nevertheless, FGF-2’s role in ALS differed from its involvement in previous diseases. A study conducted by Thau et al. used mouse *Sod1* mutant models to study the effects of FGF-2 on ALS. Surprisingly, the reduction in FGF-2 had a protective effect, as FGF-2-deficient mice exhibited delayed disease onset, improved motor performance, and prolonged survival [119]. This effect was interpreted as part of a complex interplay mechanism, where FGF-2 reduction led to the upregulation of other neurotrophic factors, including ciliary neurotrophic factor (CNTF) and GDNF [119]. A follow-up study confirmed that the effects of FGF-2 depletion in ALS mice are not isoform-specific; however, an FGF-2 isoform-dependent impact was noted on EGF gene expression in ALS muscle tissue [263].

### 4.7. Ciliary Neurotrophic Factor (CNTF)

CNTF is a GF from the Interleukin 6 (IL-6) family of cytokines [264]. It was first identified and isolated in 1980 by Manthorpe et al. from ocular tissue of chick embryos [265], hence the name, and was later found in the sciatic nerves if adult animals of leporine and murine species [266]. The CNTF polypeptide consists of 200 amino acids, weighing around 22.8 kDa [267]. Myelin-producing Schwann cells in the PNS and ocular tissue are considered the main source of CNTF, followed by astrocytes, microglia, and other glial cells in the CNS, such as oligodendrocytes [268,269]. As a member of the IL-6 family, CNTF exerts its functions through a receptor complex consisting of one binding protein (CNTF-R), along with two other proteins: gp130 and leukemia inhibitory factor receptor (LIF-R) [270]. CNTF serves as a multifunctional GF both in the CNS and PNS. For instance, CNTF is known for its pivotal role in promoting survival and maintaining the optic nerve system, especially by supporting retinal ganglion cells [168,169]. CNTF is also implicated in hippocampal neurogenesis [170] and neurogenesis in the sub-ventricular zone [171]. Moreover, the role of CNTF in astrocyte activation has been demonstrated in vivo through the lentiviral-vector-mediated delivery of CNTF into hippocampal tissue, leading to sustained astrocyte activation [172]. Importantly, this astrocyte activation, attributed to CNTF, has shown promise in terms of modifying the threshold for spreading depolarization characteristics of acute brain injuries [172]. Another important function of CNTF is promoting myelination, thereby enhancing nerve conduction [173].

Given the properties of CNTF, particularly its role as a promyelinating neurotrophic factor, numerous clinical trials in the late 1990s and early 2000s aimed to evaluate its efficacy in relation to ALS [271]. However, the systemic delivery of CNTF consistently led to dose-related adverse effects in patients, hindering the attainment of efficiency, and there were no significant differences observed between the trial groups and controls [120,121,122,123,124]. To address this limitation, a clinical trial conducted by Aebischer et al. tested the intrathecal delivery of CNTF in ALS patients [125]. This approach resulted in detectable measures of the growth factor in CSF for at least four months without the adverse effects associated with systemic delivery, highlighting the need for targeted delivery of CNTF to motor neurons [125].

Similarly, therapeutic approaches for PD have been suggested based on in vivo observations of CNTF’s neuroprotective effects on dopamine neurons [272]. Nam et al. found that transient receptor potential vanilloid 1 (TRPV1) activation on astrocytes leads to the endogenous production of CNTF [126]. This, in turn, prevents the active degeneration of dopamine neurons and results in behavioral recovery in rat models of PD.

### 4.8. Transforming Growth Factor-Beta 1 (TGF-β1)

TGF-β1 is a secreted cytokine, and it is the most abundant in mammals among three distinct isoforms of TGF-β comprising the TGF-β family [273]. TGF-β1 was first discovered in 1981 by Roberts and Sporn’s research team and later purified and characterized as a protein that is able to transform normal fibroblast cells into cancer-like cells [274,275]. TGF-β1 is synthesized in many cell types, with platelets, fibroblasts, and immune cells being its major sources [276]. In the CNS, it is produced mainly by microglia, astrocytes, neurons, and oligodendrocytes [277]. TGF-β1 is secreted in a biologically inactive or latent form, binding with latency-associated protein (LAP), and then associates with latent TGF-β-binding proteins to form a large latent complex (LLC) [278]. These complexes prevent TGF-β1 from functioning until activation occurs, either via proteolytic cleavage or non-enzymatic activation mediated via integrin or pH changes [278]. TGF-β1 signals through a hetero-tetrameric complex of serine/threonine kinases, including TGF-β receptor type 1 (TGFβR1, also known as ALK-5) and TGFβR2 [279]. Subsequently, TGF-β1 signaling is transmitted across downstream cascades, primarily via SMAD proteins [174]. In general, the functions of TGF-β1 are context-dependent and can vary based on the surrounding microenvironment. For instance, it regulates cell growth and proliferation differently in variant cell types. It promotes mesenchymal cell growth but inhibits the growth of specific epithelial cells [280]. Another form of regulating cell proliferation is through the induction of cell cycle arrest or programmed cell death, exerting a tumor-suppressing function [281]. This GF also contributes to the formation of extracellular matrix (ECM) [174], embryonic development [175], immune system modulation, mainly through the regulation of lymphocyte proliferation and differentiation [176], angiogenesis [177], and wound healing [178]. In the nervous system, TGF-β1 plays a neuroprotective role, which has been identified in different brain injuries [179]. In a murine model of focal cerebral ischemia, the upregulation of TGF-β1 has been found to reduce neurodegeneration, exert antioxidant activity, and support neurotrophic factors [180]. It has also been found to enhance synaptic plasticity, thereby affecting learning and memory. The impact of intranasally delivered TGF-β1 has been investigated in a murine model with induced status epilepticus and associated hippocampal damage [282]. It was found to cause a significant reduction in seizures and hippocampal damage, as well as improved cognition. Recent in vivo research has also found that the downregulation of TGF-β1 leads to impaired synaptic plasticity and memory deficits, which could be effectively reversed via TGF-β1 administration [181].

In the context of hNDDs, TGF-β1 has been repeatedly suggested to have a crucial role in the etiology of AD, with implications for both pathological mechanisms and potential therapeutic interventions. In animal models, impaired TGF-β1 signaling has been considered a hallmark of early-stage AD brains as it contributes to microglial activation and neuronal cell cycle reactivation, which are both implicated in AD neurodegeneration [283]. In humans, altered TGF-β1 levels have also been reported to be elevated in the CSF while reduced in the plasma of AD patients, which could serve both as a diagnostic marker and a potential target for neuroprotection [284]. Moreover, considering its anti-inflammatory properties, the reduced production of TGF-β1 has been suggested to be a risk factor for developing AD in patients that have mild cognitive impairment [285]. A genetic association study conducted by Dickson et al. suggested that specific TGF-β1 genetic variants, especially the −509 single-nucleotide polymorphism (SNP), may be associated with an increased risk of late-onset AD [127]. Based on observed high levels of TGF-β1 in healthy aging individuals and reduced TGFβR2 levels in AD patients, a hypothesis has been proposed that suggests using the first to address the latter, thereby preventing the progression of AD [286].

Similarly, while TGF-β1 levels have not proven reliable as a marker for HD severity, showing no correlation with motor dysfunction, they have demonstrated an association with cognitive impairment, particularly in the early stages of the disease [287].

In relation to PD, postmortem analysis revealed a significant elevation of TGF-β1 levels in both the ventricular CSF and striatal regions among PD patients when compared to healthy controls [288]. Elements of the TGF-β1 intracellular cascade have also been proven to play a role in PD pathology [289]. Particularly, Smad3 deficiency has been found to significantly affect the dopaminergic system in animal models, potentially contributing to the early stages of Parkinsonism through the induced catabolism of dopamine, decreased trophic and astrocytic support, and the potential induction of α-synuclein aggregation [290]. Accordingly, the modulation of the Smad3 signaling pathway has been suggested to be a possible neuroprotective approach in PD patients. Another piece of research conducted by Tesseur et al. investigated the effects of modulating the TGF-β1 signaling pathway via the upregulation of its type I receptor in a murine model of PD [128]. As a result, the local delivery of the ALK-5 receptor using an AAV vector (AAV-ALK-5) significantly reduced dopaminergic neurodegeneration, as well as motor deficits, suggesting the potential of TGF-β1 in PD therapy. Furthermore, TGF-β1 has been implicated in influencing astrocytic function, potentially contributing to PD’s pathology [291]. The reduced activity of TGF-β1 in astrocytes with the LRRK2 G2019S mutation, a common cause of familial PD, has been found to compromise their neuroprotective function [292].

Nevertheless, TGF-β1 seems to play a negative role in the regulation of neuroprotective inflammatory response in ALS as its astrocyte-specific overproduction in SOD1 mice has been found to accelerate disease progression and negatively correlate with their lifespan [129]. Moreover, three isoforms of TGF-β have been suggested as biomarkers for ALS in skeletal muscles, wherein elevated levels correlated with muscle weakness in humans and with disease progression in a murine model [130].

### 4.9. Hepatocyte Growth Factor (HGF)

HGF is a is a pleiotropic cytokine that is also called scatter factor (SF) due to its motogenic properties. It belongs to the family of scatter factors, or HGF-like factors [293]. HGF was discovered by the team of Dr Nakamura in the early 1980s and was partially purified from rat serum and named “Hepatocyte growth factor” or “Hepatotropin” in 1984 [182]. The major sources of HGF are mesenchymal cells in different tissues, fibroblasts mainly, as well as other cell types, such as endothelial cells and hepatocytes [294]. Mature HGF is typically considered a large glycoprotein with a molecular weight of approximately 82 kDa. It is derived from an inactive single-chain precursor named pro-HGF [295]. The activation process involves the proteolytic cleavage of pro-HGF, predominantly by serine proteases [296]. Pro-HGF is stored in the ECM by coupling with heparan sulfate proteoglycans (HSPGs), which contribute to the regulation of HGF bioavailability and its localized effects on cellular processes [295]. HGF primarily binds to its specific tyrosine kinase receptor known as c-Met (mesenchymal–epithelial transition factor), triggering a series of intracellular signaling cascades that regulate various cellular processes [296]. The mitogenic function was one of the first HGF functions to be noted for its promotion of cell proliferation and growth in hepatocytes [182], crucial in processes such as tissue repair, wound healing, and organ regeneration [183]. HGF is also known to induce the motility of cells. Moreover, HGF plays a pivotal role in the regulation of tissue structure and organization during development by supporting branching morphogenesis, tubulogenesis, and organogenesis, mainly in the liver, kidneys, and lungs [184]. With its anti-inflammatory properties, HGF demonstrates immunomodulatory effects that have found application in autoimmune disease therapies [185,186]. Notably, HGF administration in vivo has been observed to stimulate the production of immune-suppressive cytokines by regulatory T cells, highlighting its potential in modulating immune responses for therapeutic purposes [187]. In addition, as HGF and its c-Met receptor are involved in cell migration, 3-D morphogenesis, and survival, their dysregulation has been associated with the progression of different cancer types, and their serum levels correlate with treatment response and patient outcomes [297].

In the nervous system, HGF has neurotrophic properties and can promote the survival of neurons through its c-Met receptor [131]. It is also involved in the regeneration of injured peripheral nerves as it has been found to promote neurite outgrowth and extension in murine dorsal root ganglion cells, potentially by controlling mitochondrial activity [298]. Notably, HGF’s function goes beyond simple extension, acting as a chemoattractant that facilitates the axonal guidance of motor neurons [188]. In the long term, acute intrastriatal delivery of HGF in mice, following a 45 min stroke, resulted in long-term neuroprotection, decreased infarct volume, increased neuronal survival for up to 4 weeks, and preserved BBB integrity [299].

Regarding hNDDs, a recent study investigated the correlation between HGF levels in the CSF of AD patients and disease progression, considering the fact that different previous studies suggested links between white matter damage, HGF levels, and AD pathology [131]. As a result, demented participants showed significantly higher levels of CSF HGF compared to controls. In addition, there is a significant correlation between HGF levels and AD biomarkers, including Aβ42, pTau, and tTau, suggesting that HGF could potentially cause faster declines in cognition. Another research team led by Wright and Harding has worked since the 1990s on a series of experiments focused on developing small molecules capable of activating the HGF/MET system for AD therapy [132]. As a result, one such molecule, Dihexa, exhibited metabolic stability, BBB penetrability, and the capacity to improve cognitive function by promoting synaptogenesis in AD animal models and was suggested by the researchers to have both therapeutic and prophylactic potential in relation to AD.

Given its neurotrophic properties, the potential of HGF has also been explored for PD therapy. In an in vitro study conducted by Liu et al., the efficacy of mesenchymal stem cells (MSCs) genetically modified to express HGF, utilizing an adenovirus vector, was investigated as a therapeutic intervention for PD [300]. It was found that MSC-HGF could promote the regeneration of damaged PD cells more effectively compared to control MSCs, with the observed effects being attributed to the regulation of intracellular calcium levels [300]. However, there have only been a few studies to explore this approach for neural diseases [133], and the effects of HGF have often been perceived as integral components within the complete secretome of MSCs, working in concert with other cytokines to contribute to the overall exerted neuroprotective functions for PD therapy [301].

## 5. Current Challenges/Limitations of Using Growth Factors in Therapy for Hereditary Neurodegenerative Diseases

Despite the wide range of GFs’ therapeutic potential for different hNDDs, their practical use still faces many obstacles that require careful consideration for the development of effective and clinically applicable treatment options. To begin with, the proper dosing of GFs and choosing an appropriate delivery route have repeatedly proven challenging [302,303,304,305]. Factors such as molecule size, which affect BBB penetration [306,307], concerns about biosafety and the risk of adverse effects [308], and the invasiveness of procedures, particularly in cases requiring local instead of systemic delivery [306], all contribute to these obstacles. Moreover, as some GFs have shown a relatively short half-life in vivo, they may require frequent and sustained administration to maintain their therapeutic effects, which can also impose a challenge [302]. The versatile and pleiotropic nature of certain GFs, while beneficial in specific contexts, poses challenges related to limited specificity in therapeutic applications, potentially resulting in off-target effects [309]. Patient variability, stemming from genetic variations and individual differences in the microenvironment, further complicates the response to GF therapy [306]. Further, of course, the inherent complexity of hNDDs is a major challenge for GF therapy, as targeting a single GF may not sufficiently address the multifaceted nature of these diseases [310,311].

## 6. Conclusions

In conclusion, for hNDDs, several GFs have demonstrated efficiency in gene therapy. In HD, BDNF delivered through mesenchymal stem cells reduced striatal atrophy, alleviated anxiety, increased neurogenesis-like activity, and extended lifespan in murine models. Similarly, GDNF promoted neuronal survival and significantly enhanced motor performance in HD models. In PD, BDNF-secreting fibroblasts and GDNF-expressing vectors showed promising results in preventing neuronal loss, restoring motor function, and reducing neuroinflammation. VEGF also exhibited neuroprotective effects and improved motor function in PD models. For FRDA, BDNF prevented apoptosis in vitro and hindered neuropathology development in vivo. IGF-1 decreased disease progression in FRDA patients, though it did not entirely prevent it. In AD, IGF-1 blocked memory impairment in mice, and higher CSF VEGF levels were associated with healthier brain aging and slower cognitive decline in AD patients. In ALS, the intrathecal delivery of BDNF showed tolerance but inconclusive efficacy in clinical trials, while VEGF significantly delayed disease progression and preserved motor and neurological functions in murine models. Although CNTF did not show significant clinical benefits in subcutaneous delivery trials for ALS, localized delivery methods might be necessary for more convincing outcomes. Therefore, a profound understanding of the roles GFs play in neuronal survival, growth, and regeneration, as well as the molecular mechanisms involved in hNDDs’ pathogeneses, is needed for the development of targeted, efficient treatments that are clinically applicable. In this review, several innovative strategies in gene therapy for hNDDs have been presented while addressing key challenges in enhancing the stability and targeting of therapeutic agents like IGF-1 and BDNF through advanced delivery systems, such as viral vectors and nanoparticles. The discussed GF-based therapeutic approaches not only aim to slow disease progression but also promote neuroprotection and neurogenesis. By integrating current research with practical therapeutic applications, this review offers a comprehensive perspective that could lead to novel treatments and improved outcomes for patients with hNDDs. Provided their current main limitations and challenges are addressed, GF therapy for hNDDs holds significant promise and opportunities. Precision medicine, gene editing technologies, and combination therapies can all aid in developing efficient therapeutic strategies.

## Figures and Tables

**Table 1:** BDNF applications in therapy for different hNDDs.

Growth Factor	Disease	Type of Study	Application	Results	Ref.
BDNF	Huntington’s disease	In vivo	Investigation of the therapeutic potential of BDNF delivered through mesenchymal stem/stromal cells (MSC/BDNF) in HD murine model	Reduced striatal atrophy, alleviated anxiety, increased neurogenesis-like activity, and extended lifespan	[74]
	Amyotrophic lateral sclerosis	Phase I/II clinical trial	Intrathecal delivery of recombinant BDNF for therapy in ALS patients	Doses up to 150 microg/day were well tolerated, with reversible CNS effects at higher doses. However, study’s design and small patient sample did not allow conclusions about treatment efficacy	[75]
	Parkinson’s disease	In vivo	Investigation of the therapeutic potential of BDNF-secreting fibroblasts grafted near substantia nigra (SN) in a murine model	BDNF has shown promise in preventing neuronal loss and increasing survival of dopaminergic neurons in the SN and their projections to the striatum (ST)	[76,77]
				In post-PD-induction cases, no recovery of dopaminergic neurons in SN	[78]
	Spinocerebellar ataxia type 1	In vivo	Intraventricular delivery of recombinant BDNF in a murine model	Delayed the onset of motor impairments and neuronal degeneration, and ameliorate them in the post-symptomatic stage. However, no changes in gene expression were noticed in Purkinje cells	[79]
	Friedreich’s ataxia	In vitro	Investigation of the protective effects of adipose stem cell-conditioned medium containing BDNF against oxidative stress in FA cells	BDNF prevented apoptosis triggered by FXN gene knockdown	[80]
		In vivo	Intracerebral delivery of herpes-viral vector carrying a BDNF gene in a murine model	Prevented apoptosis and hindered the development of neuropathology characteristic of FRDA	[81]

**Table 2:** GDNF applications in therapy for different hNDDs.

GDNF	Parkinson’s disease	In vivo	Intravenous injections of GDNF-expressing macrophages in a murine model	Promising results for both early and late stages of the disease, including enhanced brain tissue integrity, restoration of most motor functions, sustained therapeutic effects, reduced neuroinflammation, and diminished α-synuclein aggregation	[82]
			Intrastriatal injection of rAAV-GDNF in the common marmoset monkey (*Callithrix jacchus*)	Improved protection of dopaminergic neurons and higher dopamine levels	[83]
			Intracerebral delivery of AAV2-hGDNF in rats	Generally favorable safety profile. However, none have provided robust and consistent evidence for clinical efficiency due to challenges including dosing, delivery route, and immune response	[84,85,86,87,88,89,90,91]
			Intracerebral delivery of AAV2-hGDNF in rhesus macaques		
		Several clinical trials	Putaminal infusion of AAV2-GDNF in patients with advanced PD		
			Bilateral intraputaminal infusion of recombinant GDNF in PD patients		
	Huntington’s disease	In vivo	Intrastriatal injection of AAV-GDNF in a murine model	Promoted neuronal survival, and significantly enhanced performance in motor tasks and neurological assessments, which was also supported by histological analyses showing higher neuronal counts in the targeted brain regions	[92,93]
			Intrastriatal injection of GDNF-secreting neural stem cells (NSCs-GDNF) in a murine model		

**Table 3:** IGF-1 applications in therapy for different hNDDs.

IGF-1	Alzheimer’s disease	In vivo	Intracerebroventricular injection of rAAV-IGF1 in AD mice	Blocked memory impairment, accordingly, could serve as a potential strategy against neuronal damage and memory loss in AD	[94]
	Amyotrophic lateral sclerosis	Phase III clinical trial	Subcutaneous delivery of human recombinant IGF-1 for 2 years in ALS patients	No significant improvement in outcomes, compared to the initial point, and IGF-1 therapy was found to be non-beneficial for this condition	[95]
		Phase II clinical trial	Intravenous delivery of an IGF-1 mimetic, with a longer half-life, called BVS857 in ALS patients	Provoked an immune response after 12 weeks, with no significant improvement in strength or function of the muscles	[96]
	Huntington’s disease	Ex vivo	Investigating the effects of activating IGF-1/insulin signaling pathway in lymphoblasts derived from HD patients	Improved mitochondrial and metabolic function, as well as energy production, highlighting the role of IGF-1 in HD lymphoblasts	[97]
	Friedreich’s ataxia	Clinical pilot study	Subcutaneous delivery of IGF-1 in FRDA patients for 12 months	Decreased disease progression during therapy, some patients exhibited high scores on the ataxia scale towards the end of the study. IGF-1 was found to significantly decrease FRDA progression but did not entirely prevent it	[98]
	Ataxia telangiectasia	Cross-sectional observational study	Investigation of the correlation between clinical neurological data, including IGF-1 levels, with extracerebellar neuroimaging findings in AT patients	Established correlation between the deficiency of IGF-1 axis and elevated ataxia scores, coupled with severe neurodegeneration	[99]

**Table 4:** Applications of VEGF-A in therapy for different hNDDs.

VEGF-A	Alzheimer’s Disease	Prospective longitudinal study	Examining how VEGF levels relate to brain aging over time across different cognitive statuses and investigating VEGF interactions with established AD biomarkers.	Higher CSF VEGF levels were linked to healthier brain aging. VEGF’s protective effects were strongest with heightened AD markers, indicating potential benefits for early AD stages	[100]
	Amyotrophic lateral sclerosis	In vivo	Intrathecal injection of AAV-VEGF in a murine model	Significant delay of disease, preserved motor and neurological functions, and a longer life span	[101]
	Parkinson’s disease	In vivo	Intrastriatal injection of AAV-VEGF in a murine model	Neuroprotective effects on dopaminergic neurons, and positive behavioral outcome	[102,103]
			Intrastriatal infusion of hVEGF-secreting cells (baby hamster kidney-VEGF) in a murine model		
		In vitro/in vivo	Investigation of cystatin C effect following intrastriatal injections in a murine model and its influence on VEGF secretion	VEGF elevation in the targeted area preserved dopaminergic neurons and promoted angiogenesis in vitro	[104]
			Non-viral intrastriatal delivery of VEGF gene to dopaminergic neurons using a polymeric gene carrier in a murine model	Preserved motor function, with no loss of dopaminergic neurons in the targeted area. Suppression of both microglial activation and apoptosis was proposed to contribute to the overall therapeutic effect of VEGF	[105]

**Table 5:** Applications of NGF in therapy for different hNDDs.

NGF	Alzheimer’s Disease	Phase I clinical trial	Intracerebral injections of AAV2-NGF in AD patients	Proven feasibility, good tolerance, and long-term production of biologically active NGF (up to 7 years)	[106]
		Phase II clinical trial		No changes in cognition or clinical outcomes, attributed to the delivery route	[107]
		Phase I clinical trial	Either ex vivo NGF gene delivery using genetically modified fibroblasts or intracerebral injections of AAV2-NGF in AD patients	Conserved ability of degenerating brain regions to interact with the delivered GF, evidenced by axonal sprouting and activation of cell signaling, with no NGF-related reported adverse effects	[108]
		Phase I clinical trial	Encapsulated cell bio-delivery of NGF (NGF-ECB), intracerebral injections in AD patients	Good tolerance in all patients, with three patients showing less brain atrophy and higher CSF cholinergic markers	[109,110].
	Huntington’s disease	In vivo	Intracerebral injections of recombinant purified NGF in a murine model	Considerable positive effect on cognitive function, elevated cholinergic markers were noticed, along with restored neurogenesis in the hippocampus and enhanced spatial working memory	[111]
	Parkinson’s disease	In vivo	Intrastriatal injections of genetically modified bone marrow stromal cells expressing NGF (BMSC-NGF)	Induced neurogenesis and a significant improvement in rotational behavior	[112]
	Amyotrophic lateral sclerosis	Retrospective, observational study	Investigating the effect of mouse nerve growth factor (mNGF) combined with riluzole (an FDA-approved drug for ALS) on ALS patients	Although proven safe and well tolerated, the treatment did not result in significant clinical changes in ALS progression	[113]

**Table 6:** Application of FGF-2 in therapy for different hNDDs.

FGF-2	Alzheimer’s Disease	In vivo	Subcutaneous delivery of recombinant FGF-2 in a murine model	Improved spatial memory, elevated levels of astrocytes in the hippocampus, and potential modulation of inflammatory responses	[114]
		In vivo	Intracerebral injections of AAV2-FGF-2 in a murine model	Improved spatial learning and long-term potentiation	[115]
		Detailed summary of in vitro and in vivo studies exploring FGF-2 therapeutic potentials and effects for AD			[116]
	Parkinson’s disease	In vivo	Investigating the effect of silencing miR-497-5p (an FGF-2 repressor) in a murine model	FGF-2- mediated improvements in motor symptoms, reduction in apoptosis, and stimulation of autophagy	[117]
		In vitro	Investigating FGF2 influence on the release of extracellular vesicles (EVs) in hippocampal neurons, and its relevance to PD	Enhanced release of EVs enriched with Rab8b and Rab31, suggesting that FGF2-induced Rab enrichment in EVs could play a role in molecular mechanisms related to non-motor symptoms in PD, such as hearing loss and dementia.	[118]
	Amyotrophic lateral sclerosis	In vivo	Investigating effects of FGF-2 deficiency in a murine model of ALS	FGF-2-deficient mice exhibited delayed disease onset, improved motor performance, and prolonged survival, as FGF-2 reduction led to the upregulation of other neurotrophic factors, including CNTF and GDNF	[119]

**Table 7:** Applications of CNTF in therapy for different hNDDs.

CNTF	Amyotrophic lateral sclerosis	Several clinical trials	Subcutaneous delivery of recombinant CNTF in ALS patients	No significant differences observed. Common major adverse effects included injection site reactions, cough, reactivation of herpes simplex virus (HSV1) labialis/stomatitis nausea, anorexia, weight loss, and increased salivation	[120,121,122,123,124]
		Phase I clinical trial	Intrathecal implantation of polymer capsules containing genetically engineered baby hamster kidney cells releasing human CNTF in ALS patients	Detectable measures of CNTF in CSF for at least four months, without the adverse effects associated with systemic delivery	[125]
	Parkinson’s disease	In vivo	Investigating the effect of CNTF through activating transient receptor potential vanilloid 1 (TRPV1) on astrocytes in a murine model	Prevented the active degeneration of dopamine neurons and resulted in behavioral recovery in PD rat models	[126]

**Table 8:** Applications of TGF-β1 in therapy for different hNDDs.

TGF-β1	Alzheimer’s disease	Genetic association study	Investigation of the correlation between TGF-β1 variants and AD	Potential association between specific TGF-β1 genetic variants, especially the −509 single-nucleotide polymorphism (SNP) and increased risk of late-onset AD	[127]
	Parkinson’s disease	In vivo	Intracerebral injection of an AAV vector expressing type I receptor of TGF-β1 (AAV-ALK-5) in a murine model	Significant reduction in dopaminergic neurodegeneration and motor deficits	[128]
	Amyotrophic lateral sclerosis	In vivo	Investigation of TGF-β1 role in the regulation of neuroprotective inflammatory response in a murine model of ALS with astrocyte-specific overproduction	Overproduction of TGF-β1 accelerated disease progression and negatively correlated with lifespan	[129]
		Observational validation study	Investigating the potential role of TGF-β1 isoforms as biomarkers of ALS progression	Elevated TGF-β1 levels correlated with muscle weakness in humans, and with disease progression in a murine model	[130]

**Table 9:** Applications of HGF in therapy for different hNDDs.

HGF	Alzheimer’s disease	Observational cohort study	Investigation of the associations between CSF HGF levels, AD biomarkers, and cognitive function.	Significantly higher levels of CSF HGF in demented participants and a significant correlation between HGF levels and AD biomarkers, including Aβ42, pTau, and tTau	[131]
		In vivo studies	Developing and testing small molecules capable of activating the HGF/MET system for AD therapy in AD animal models	Dihexa (one of the developed small molecules) exhibited metabolic stability, BBB penetrability, and the capacity to improve cognitive function by promoting synaptogenesis	[132]
	Parkinson’s disease	In vitro	Investigation of the efficacy of MSCs expressing HGF (MSC-HGF) using an adenovirus vector in a PD cell model	Promoted regeneration of damaged PD cells, through regulation of intracellular calcium levels	[133]

**Table 10:** Characteristics of different GFs.

Growth Factor	Classification	Discovery	Size	Primary Source	Receptors	Functions
BDNF	Neurotrophins	Discovered in 1982 in pig brains	28–32 kDa	Neurons and glial cells	TrkB (high-affinity) and p75NTR (low-affinity)	Neuronal survival [134], neuroplasticity and synaptic plasticity [135], neurogenesis and synaptogenesis [136], cognitive function [137], dendritic branching [138], regulation of gene expression [135], and modulation of excitatory and inhibitory neurotransmitter profiles
GDNF	Transforming growth factor beta (TGF-β) superfamily	Discovered in 1993, in rat B49 glial cell lines	24 kDa	Glial cells	Receptor complex of Ret receptor tyrosine kinase and one of a number of glycosylphosphatidylinositol (GPI)-anchored cell surface proteins GFRα1–4	Potent survival factor for neurons [139,140]. Control migration and neuronal differentiation in the enteric nervous system in vivo [141]. Regulation of ureteric budding and branching [142], and spermatogenesis regulation [143]
IGF-1	Insulin-like growth factor family	Discovered in 1976, in human serum	7.6 kDa	Hepatocytes mainly, but also produced in the brain stem, cerebellum, cerebral cortex and the striatum	Tyrosine-kinase IGF-1 receptor (IGF-1R)	Growth stimulation [144], cellular proliferation and apoptosis [145], immunomodulatory functions [146], as well as CNS-related functions, such as neurogenesis, angiogenesis, neuroprotection, myelination, modulation of neuroinflammatory response, and neuroplasticity
VEGF-A	Vascular endothelial growth factor family	Discovered in 1980s and was identified as secretions from tumor cells	20–27 kDa	Endothelial cells engaged in angiogenesis process and maintaining barrier permeability, glial cells, and neurons	VEGF Receptors (VEGFRs), mainly VEGFR1 and VEGFR2	Enhancement of synaptic plasticity, influencing memory and learning processes [147,148]. Support neuronal survival in vivo through involvement in angiogenesis and reducing neuroinflammation [149]. Upregulation of VEGF-A may serve in counteracting neuronal damage and enhance survival pathways [150]. Enhancement of axonal growth [151], and activation of Schwann cells [152].
NGF	Neurotrophin family	Discovered in 1957 from mouse sarcoma 180. (a Nobel Prize-winning discovery)	130 kDa	Neurons and glial cells	p75 neurotrophin receptor (p75NTR) for proNGF, and tyrosine kinase A receptor (TrKA) for NGF	Modulation of neuronal growth, proliferation, activation, and survival [153], regulation of sensory neurons differentiation [154], perception [155], axonal target innervation [156], cholinergic neurons maintenance [157], and synaptic plasticity, affecting learning and memory abilities [158], with lower levels associated with depression [153].
FGF-2	Fibroblast growth factor family	Discovered by contributions from various researchers during the 1970s and 1980s	Two isoforms: 18 kDa (low molecular weight), and 21–34 kDa (high molecular weight)	Astrocytes	An isoform of the FGF receptor (FGFR1–4) depending on the cell type	Potent mitogen for cell proliferation [159], promotes angiogenesis [160], tissue repair and regeneration [161], and cell activation and migration, impacting inflammation, wound healing, and potentially cancer progression [162,163]. Neurotrophic effects: promoting neuronal survival [164], neurogenesis [165], axonal growth, dendritic arborization [166], and synaptic plasticity, influencing cognition and memory [167].
CNTF	Interleukin 6 family	Discovered in 1980 from ocular tissue of chick embryos, and later found in the sciatic nerves in adult animals of leporine and murine species	22.8 kDa	Myelin-producing Schwann cells, in PNS and ocular tissue, followed by astrocytes, microglia and oligodendrocytes	Receptor complex: CNTF-R with gp130 and leukemia inhibitory factor receptor (LIF-R)	Survival and maintenance of the optic nerve system, especially, by supporting retinal ganglion cells [168,169]. Hippocampal and sub-ventricular neurogenesis [170,171]. Astrocyte activation [172]. Promoting myelination and enhancing nerve conduction [173].
TGF-β1	Transforming growth factor-beta family	Discovered in 1981 and was later characterized as a protein able to transform normal fibroblast cells into cancer-like cells	25 kDa	Platelets, fibroblasts, and immune cells. In the CNS: microglia, astrocytes, neurons, and oligodendrocytes	Hetero-tetrameric complex of serine/threonine kinases including TGF-β receptor type 1 (TGFβR1, also known as ALK-5) and TGFβR2	Regulation of cell growth and proliferation, contributing to the formation of extracellular matrix (ECM) [174], embryonic development [175], immune system modulation, regulation of lymphocyte proliferation and differentiation [176], angiogenesis [177], and wound healing [178]. In the nervous system: neuroprotective role [179]. Reducing neurodegeneration, exerting antioxidant activity, and supporting neurotrophic factors [180]. Enhancing synaptic plasticity, affecting learning and memory [181].
HGF	Family of scatter factors, or HGF-like factors	Discovered in the early 1980s and was partially purified from rat serum	82 kDa	Mesenchymal cells in different tissues, mainly fibroblasts, and other cell types such as endothelial cells and hepatocytes	Specific tyrosine kinase receptor known as c-Met (mesenchymal–epithelial transition factor)	Mitogenic function by promoting cell proliferation and growth in hepatocytes [182], tissue repair, wound healing, and organ regeneration [183]. Inducing cells’ motility. Regulation of tissue structure and organization during development, by supporting branching morphogenesis, tubulogenesis, and organogenesis [184]. Anti-inflammatory properties and immunomodulatory effects [185,186,187]. In the nervous system: neurotrophic properties and promoting the neurons’ survival [131]. HGF’s function goes beyond simple extension, acting as a chemoattractant that facilitates the axonal guidance of motor neurons [188].
